# A Simulation of Air Lasing Seeded by an External Wave in a Femtosecond Laser Filament

**DOI:** 10.3390/s23208364

**Published:** 2023-10-10

**Authors:** Tao Zeng, Ya Gui, Yuliang Yi, Nan Li, Zhi Zhang, Jiewei Guo, Binpeng Shang, Lanjun Guo

**Affiliations:** 1 Chongqing Key Laboratory of Micro & Nano Structure Optoeletronics, School of Physical Science and Technology, Southwest University, Chongqing 400715, China; taozeng@swu.edu.cn (T.Z.);; 2 Chongqing Vocational College of Transportation, Chongqing 402247, China; 3 Tianjin Key Laboratory of Micro-Scale Optical Information Science and Technology, Institute of Modern Optics, Nankai University, Tianjin 300350, China

**Keywords:** femtosecond laser filamentation, air laser, spatial distribution, nitrogen ion density

## Abstract

Air lasers induced by femtosecond laser filaments play an important role in remote sensing applications. Few studies have been dedicated to the spatial distribution of external-seeded air laser radiation in the laser filament based on the numerical simulation method, which can pave the way to understanding the mechanism of the external-seeded air lasing process during filamentation. In this study, numerical simulations of the propagation of an air laser seeded by an external plane wave with a wavelength of 391 nm during femtosecond laser filamentation were performed. The results indicated that the air laser’s beam intensity distribution varies from a ring pattern to a donut pattern when the filament length and nitrogen ion density are raised as a result of the defocusing and lasing effects of the filament plasma. Here, the ring pattern is formed by several thin rings, while the donut pattern refers to a notably thicker, ring-like structure. In addition, it has been demonstrated that the air laser’s beam power would increase exponentially versus the filament length and the nitrogen ion density. The knowledge about the angular distribution of air lasing could be important for optimizing the detection geometry of the LIDAR setup, including the view angle and the size of the collecting optical component.

## 1. Introduction

Air lasing based on femtosecond laser filaments has attracted extensive interest during the last decade due to its promising applications for the remote sensing of pollutants in the atmosphere [1,2,3,4]. Since an extremely intense light field could be confined in the laser filament core over an extended propagation distance, various substances in the air such as gases, dusts, and aerosols would be ionized [5,6,7,8]. A fluorescence with a characteristic spectrum would be generated and then enhanced during the propagation in the filament, which is called the amplified spontaneous emission (ASE). This unique phenomenon has been widely reported, referred to as an air laser [9,10,11,12,13,14].

Self-seeded air lasers with different profiles have been investigated. A high-gain air laser in the backward direction has been achieved at a wavelength of 845 nm by the lasing of oxygen atoms. A donut shape was demonstrated with a divergence angle of 40 mrad [11]. UV air laser pulses at 337 nm and 357 nm were generated from the second-positive-band transitions of N_2_ in an argon-nitrogen gas mixture. A roughly super-Gaussian profile with a divergence angle of 1.6 mrad was reported [15]. Additionally, gas pressure has a significant role in the air laser’s intensity and spatial profile. The profile of the self-seeded lasing from nitrogen ions changes from a Gaussian pattern to an outer ring structure with the increase of gas pressure. Moreover, the outer ring is better seen with an increase in gas pressure [16].

Meanwhile, the lasing action could also be realized by a filament-induced seed light. The lasing action of N_2_^+^ was activated by the injection of a seed pulse around 400 nm. The population inversion in molecular N_2_ ions was induced by femtosecond laser filamentation at 800 nm [17]. A remote, harmonic-seeded, switchable multiwavelength laser has been demonstrated in the forward direction in air by intense midinfrared femtosecond laser pulses [18]. Moreover, the generation of a remote air laser with a donut-shaped beam pattern was realized using an 800 nm femtosecond laser pulse and seeded by white light. The pump laser plays two roles: establishing the population inversion of the ions in the filament and generating the supercontinuum seed light [19].

The air lasing signal at 391 nm evolving from a Gaussian mode into a cylindrical vector beam mode in the N_2_^+^ gain medium has been studied using the numerical simulation method. Moreover, the time dependence of the population distribution in the ground and exited states of N_2_^+^ was neglected [20]. A collective emission in ionized nitrogen molecules triggered by a coherent seed pulse has been investigated by the theoretical method. The theoretical predictions of the forward emission at 391 nm as a function of the nitrogen gas pressure are in good agreement with the experimental results [21]. Nevertheless, the mechanism of the ring or donut pattern spatial profile of the air laser is still not quite clear. In addition, the investigation of the ring or donut air laser pattern seeded by an external wave in the filament has not yet been considered based on the numerical simulation method.

In this article, the spatial intensity distribution of an external-seeded air laser with a wavelength of 391 nm propagating through the femtosecond laser filament in ambient air was investigated based on the numerical simulation method. The results revealed that the air laser’s intensity distribution after the filament was changed from a ring pattern to a donut pattern by raising the filament length or nitrogen ion density, as a result of the plasma’s defocusing and lasing effects. In addition, it has been demonstrated that the gain coefficient of the air laser inside the filament increases with both the filament length and the nitrogen ion density. Therefore, the air laser’s beam power increases exponentially versus the filament length and the nitrogen ion density. The knowledge about the angular distribution of air lasing could be important for optimizing the detection geometry of the LIDAR setup, including the view angle and the size of the collecting optical component, which would be valuable for the remote sensing of air pollutants based on femtosecond laser filaments.

## 2. Theoretical Method

Since we focused exclusively on the spatial distribution of the air laser, the propagation of the seed light in the filament plasma could be carried out based on the following nonlinear wave equation [22]:

(1) $$2ik_{0}\frac{\partial A}{\partial z}+\left(\frac{\partial^{2}}{\partial x^{2}}+\frac{\partial^{2}}{\partial y^{2}}\right)A+2\frac{k_{0}{}^{2}}{n_{0}}n_{\mathrm{nl}}A-ik_{0}\alpha A=0$$

where *A* denotes the envelope of the wave beam amplitude. $k_{0}$ is the wave number of the laser beam. $n_{0}$ represents the refractive index of air. $n_{\mathrm{nl}}$ denotes the nonlinear refractive index induced by the filament plasma’s defocusing effect, which is directly proportional to the nitrogen ion density inside the plasma.

In this work, a plane continuous wave with a wavelength of 391 nm was chosen to be the seed light for the lasing action, which corresponds to the vibrational transition of the N_2_^+^ first negative (0,0) band ($B^{2}\sum_{u}{}^{+}\to X^{2}\sum_{g}{}^{+}$) [23]. In the experiment, the seed light could be generated by the second harmonic of an input laser beam at a central wavelength of around 800 nm passing through a typeⅠbeta barium borate (BBO) crystal [4]. Rotating the azimuthal orientation of the crystal and inserting a narrowband interference filter, the output laser beam with the desired wavelength of 391 nm can be realized efficiently.

The lasing effect could be realized by the excitation of all three main components of air with a particular pump femtosecond laser wavelength, including nitrogen, oxygen, and argon. The air lasing based on the ionic nitrogen molecules has attracted particular attention due to its fruitful observed effects and high concentration in air. Pumped by the femtosecond laser pulses at 800 nm, nitrogen ions give rise to a series of narrow emission lines at 391.4 nm, 427.8 nm, and 471.2 nm. The N_2_^+^ lasing at 391 nm associated with the $B^{2}\sum_{u}{}^{+}\to X^{2}\sum_{g}{}^{+}$ emission induced by the femtosecond laser filament is noteworthy among the air lasing emission lines, ensuring that the population transferring process from the ground state could be monitored. Moreover, the air lasing at 391 nm has been commonly observed in experiments under certain conditions.

It is worth mentioning that a stationary, homogeneous, and isotropic plasma model was adopted without considering the temporal variation. The pulse duration of 391-nm lasing emission generated from the filament plasma was measured to be several ps [24,25,26] or several tens of ps [27] by using the pump-probe method. The filament plasma lifetime was measured to be within a few ns by using the electric conductivity measurement method [28,29]. Here, the filament plasma lifetime refers to the decay time of the plasma density, including both the nitrogen and oxygen ions, due to electron-ion recombination. In addition, since many efforts have been dedicated to prolonging the lifetime of the filament plasma channel, the corresponding plasma channel lifetime extends from several ns to microsecond levels [30,31,32,33,34,35,36]. As the oxygen ion density can exist for a long time after the laser pulse, the decay time of the N_2_^+^ ion density would be less than the filament plasma lifetime. The N_2_^+^ lasing at 391 nm was associated with the $B^{2}\sum_{u}{}^{+}\to X^{2}\sum_{g}{}^{+}$ emission induced by the femtosecond laser filament. The lifetime of the $B^{2}\sum_{u}{}^{+}$ level state of the nitrogen ions induced by electron bombardment was measured to be around 59 ns [37,38]. Hence, the decay time of the nitrogen iron density induced by the electron-ion recombination could be estimated to be on the nanosecond time scale, which is much slower than the 391-nm lasing emission pulse duration in the picosecond range [24,25,26,27]. Therefore, the nitrogen ion density is considered to be conserved. This has also been claimed in recent simulation studies, while the simplified method has also been adopted [20,21].

The temporal aspect of the emitted light field was not considered in the simulation. The validity of this simplified model for the nonlinear propagation of a laser pulse has been demonstrated in previous studies [39,40]. Thus, temporal effects, including self-steeping and the group-velocity dispersion effect, were neglected in our simulation model. Actually, this kind of simplification would significantly influence the temporal dynamics of the air laser. However, to a lesser extent, it would influence the spatial evolution of the air laser, in principle. Typically, the air laser radius inside the filament is less than 100 μm. The corresponding longitudinal scale of spatial transformation $2\pi a^{2}/\lambda$ is less than 8 cm. The second-order group velocity dispersion coefficient $k^{\prime \prime }$ for air is 0.2 fs^2^/cm [41]. For the air laser pulse with pulse duration at the picosecond timescale [24,25,26,27] the characteristic dispersion length scale $\tau^{2}/k^{\prime \prime }$ of the temporal changes is much longer than the spatial transformation scale. Therefore, the spatial effects develop much faster than the temporal effects [41].

Different temporal slices of the seed pulse showed differently pronounced population inversions. Actually, the simulation model of the air laser propagating in the filament in this study was based on the precondition that the nitrogen ions’ lifetime was much longer than the air lasing pulse duration [24,25,26,27]. Therefore, there exists a very slight difference in the population inversions for different slices of the air lasing pulse, which could be neglected in the simulation.

The nitrogen ions induced by femtosecond laser filaments can be considered as the gain medium during the propagation of the seed light inside the filament plasma. α refers to the gain coefficient, which is proportional to both the nitrogen ion density *N*_e_ and the stimulated emission cross-section σ written as follows [42,43]:

(2) $$\alpha =N_{e}\sigma$$

The vibrational transition at 391 nm corresponds to a transition cross section σ of $14.8\times 10^{-18}{\;\mathrm{cm}}^{2}$ [23]. In our simulation, the nitrogen ion density distribution is based on the following empirical equation:

(3) $$N_{e}(r,l)=N_{e,\max}\exp\left[-\left(\frac{r^{2}}{{r_{0}}^{2}}+\frac{l^{2}}{{l_{0}}^{2}}\right)\right]$$

where *r* and *l* are the spatial location variables in the cylindrical coordinate system along the radius direction and longitudinal direction, respectively. The origin is set at the center of the filament plasma. *r*_0_ and *l*_0_ denote the waist radius in the cross-section and the half-waist length along the longitudinal direction, respectively. *r*_0_ is set to be 80 μm, corresponding to a filament core diameter of 160 μm. $N_{e,\max}$ is the maximum nitrogen ion density in the filament, the value of which varies from 10^15^ cm^−3^ to 10^18^ cm^−3^, generally, depending on the input femtosecond laser parameters and focusing conditions.

The plasma density distribution in the filament plasma channel along the radial direction can be fitted by a Gaussian function [44]. Moreover, the plasma density distribution along the longitudinal direction could also be close to a Gaussian profile without considering the refocusing phenomenon of the laser filament [45]. Therefore, the distribution of plasma density based on Equation (3) was taken to describe the filament plasma channel. In addition, the axicon has been commonly used in the process of filamentation for elongating the filament length, which would be quite beneficial for atmospheric applications based on the filamentation. The plasma density distribution generated from the Bessel laser beam along the longitudinal direction also obeys a Gaussian profile [46].

Overall, the applicability of the simulation model is clarified as follows: Firstly, the simulation model with a stable and conserved nitrogen ion density distribution is applicable for the seed pulse at 391 nm. The external seed pulse at 391 nm could be generated by the second harmonic of an input femtosecond laser pulse at an 800 nm wavelength passing through the BBO crystal at a slightly rotating angle. The corresponding harmonic seed pulse duration is a few tens or hundreds of femtoseconds [4,17]. Moreover, the lasing emission at 391 nm induced by the filament plasma could also serve as the seed pulse with a pulse duration of several ps or several tens of ps [24,25,26,27]. Pumped by the intense femtosecond laser pulses at 800 nm, the nitrogen ions could be populated in B and X states in the filament plasma with a two-level system. The population decay of the two-level system due to electron-ion inelastic collisions and electron-ion recombination occurs on a nanosecond timescale, which is several orders of magnitude larger than the seed pulse duration. Therefore, the simulation model with a stable and conserved nitrogen ion density distribution is applicable. Secondly, the nitrogen ion density distribution model is applicable for a single-filament plasma channel in the presence of external focusing by a lens or an axicon. The nitrogen ion density distribution in the filament plasma channel focused by a lens or an axicon along both the radial and the longitudinal direction would be close to a Gaussian profile [44,45,46]. Thirdly, the neglect of the temporal aspect of the seed pulse in the simulation model is also applicable in this study, as clarified in the above discussion.

## 3. Results and Discussion

**Figure 1:**
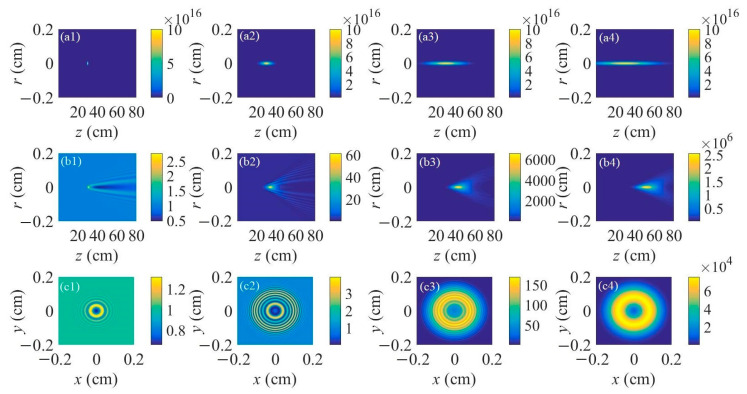
(**a1**–**a4**) Spatial distributions of the filament-induced nitrogen ion density versus propagation distance. (**b1**–**b4**) Spatial distributions of the air laser’s intensity versus propagation distance. (**c1**–**c4**) Transverse patterns of air laser intensity distribution on the cross-section at a propagation distance of 30 cm after the right end of the filament. The filament lengths are (**a1**,**b1**,**c1**) 1 cm, (**a2**,**b2**,**c2**) 10 cm, (**a3**,**b3**,**c3**) 30 cm, and (**a4**,**b4**,**c4**) 50 cm, respectively.

Different filament lengths were chosen for the lasing action during the propagation of an external-seeded plane wave inside of the filament plasma. Figure 1a1–a4 represents the spatial distributions of the nitrogen ion density for the filament lengths of 1 cm, 10 cm, 30 cm, and 50 cm, respectively. The peak nitrogen ion density is fixed at 10^17^ cm^−3^, which is a common value pumped by the input femtosecond laser pulse with a central wavelength of 800 nm and a pulse energy of several mJ under a relatively tight focusing condition. The corresponding spatial intensity distributions of the forward air lasers seeded by the plane continuous wave with a normalized initial beam intensity for different filament lengths are displayed in Figure 1b1–b4. The centers of the filament plasma are all set at *z* = 30 cm. The results indicate that the gain effect mainly occurs inside the filament plasma. The air laser diverges during the free space propagation after the filament plasma. It also appears that with the increase of the filament length, the peak intensity of the air laser increases heavily. The intensity distributions on the cross-section of the air laser’s beam are registered at a distance of 30 cm after the right end of the filament, as shown in Figure 1c1–c4 for different filament lengths. Figure 1c1–c3 shows a ring structure distribution of the air laser’s intensity. With the increase of the filament length, the number of outer rings increases. Moreover, the outer rings get denser with a longer filament length, indicating that the separation between the two adjacent outer rings gets smaller. Interestingly, when the filament length reaches 50 cm, a donut pattern of the air laser’s cross-sectional intensity distribution occurs as shown in Figure 1c4, resulting from the fact that both the number and density of the outer rings become extremely large with a relatively long filament.

**Figure 2:**
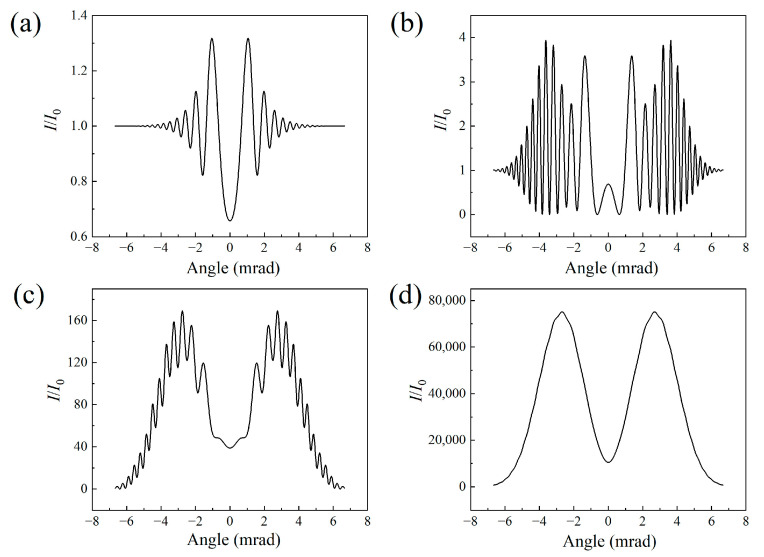
Angular distributions of the air laser’s intensity for filament lengths of (**a**) 1 cm, (**b**) 10 cm, (**c**) 30 cm, and (**d**) 50 cm.

Figure 2 depicts the signal intensity of the air laser as a function of the divergent angle for different filament lengths. The results further confirm the change from a ring structure pattern to a donut-shaped pattern of the air laser indicated in Figure 1c1–c4. The ring structure mainly results from the defocusing effect of the filament plasma. The air laser diffracts more significantly as the filament length increases. Since the outer rings get denser due to the strong diffraction effect, the adjacent rings combine with each other and spikes occur, as shown in Figure 2c. After further raising of the filament length, the spikes merge into the profile of the air laser’s signal, leading to a smoothly distributed, donut-shaped pattern with a divergence angle of 2.7 mrad, as indicated in Figure 2d.

**Figure 3:**
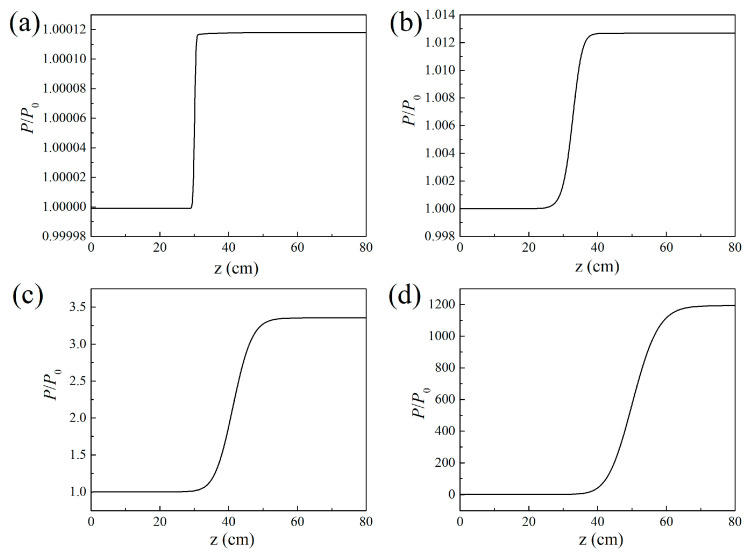
Air laser beam power along the propagation direction for the filament lengths of (**a**) 1 cm, (**b**) 10 cm, (**c**) 30 cm, and (**d**) 50 cm.

**Figure 4:**
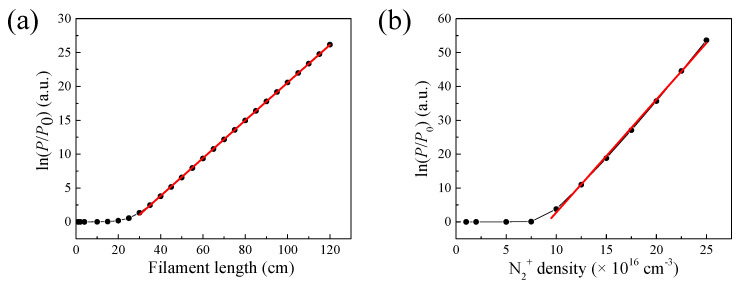
(**a**) $\ln(P/P_{0})$ as a function of the filament length (black line) and the linear fitting with the filament length larger than 30 cm (red solid line). The peak nitrogen ion density is set to be 10^17^ cm^−3^. *P*_0_ and *P* denote the air laser’s beam power before and after the filament region, respectively. (**b**) $\ln(P/P_{0})$ as a function of the peak nitrogen ion density (black line) and the linear fitting with the nitrogen ion density larger than 10^17^ cm^−3^. The filament length is set to be 40 cm.

The air laser’s beam power along the propagation direction was interpreted as depicted in Figure 3. The results indicate that the air laser experiences a gain effect inside the filament plasma region and remains constant after that. The output air laser’s beam power increases heavily versus the filament length. In order to quantitatively investigate the relationship between the gain effect and the filament length, the result of the gain coefficients varying with the filament length is displayed in Figure 4a. The gain coefficient could be reflected by $\ln\left(P/P_{0}\right)$. *P*_0_ and *P* denote the air laser’s beam power before and after the filament plasma region, respectively. The peak nitrogen ion density is set to be 10^17^ cm^−3^. When the filament length is larger than 30 cm, the relationship between $\ln\left(P/P_{0}\right)$ and the filament length could fit well linearly. This implies that the air laser’s beam power increases exponentially versus the filament length.

**Figure 5:**
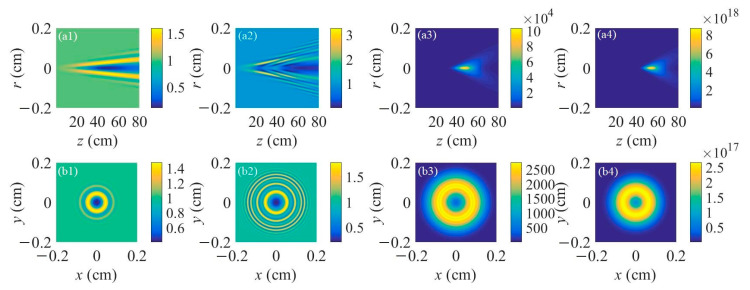
(**a1**–**a4**) Spatial distributions of air laser intensity versus propagation distance. The filament length is set to be 40 cm. (**b1**–**b4**) Transverse patterns of air laser intensity distribution on cross-sections at a propagation distance of z = 80 cm. The peak nitrogen ion densities are (**a1**,**b1**) 10^16^ cm^−3^, (**a2**,**b2**) 5 × 10^16^ cm^−3^, (**a3**,**b3**) 10^17^ cm^−3^, and (**a4**,**b4**) 2 × 10^17^ cm^−3^, respectively.

In the next step, the air laser’s intensity for different peak nitrogen ion densities was investigated. The results are shown in Figure 5. Figure 5a1–a4 displays the air laser’s intensity distribution along the propagation distance for the peak nitrogen ion density of 10^16^ cm^−3^, 5 × 10^16^ cm^−3^, 10^17^ cm^−3^, and 2 × 10^17^ cm^−3^, respectively. The filament length is set to be 40 cm. With the increase of the nitrogen ion density, the output air laser’s intensity increases significantly due to a larger gain coefficient inside the filament plasma. Figure 5b1–b4 depicts the intensity distribution of the air laser on cross-sections at a propagation distance of 80 cm corresponding to Figure 5a1–a4, respectively. It appears, just as in the results in Figure 1, that the number and density of the outer rings increases with nitrogen ion density. When the peak nitrogen ion density reaches 2 × 10^17^ cm^−3^, a donut-shaped pattern occurs, as indicated in Figure 5b4.

It was demonstrated that by increasing the gas pressure, a donut-shaped beam intensity pattern occurs for the self-seeded air laser pulse propagating after the laser filament in the experiment. The air laser seed pulse is a second harmonic wave with a central wavelength of around 400 nm induced by the femtosecond laser filament. The nitrogen ion density inside the filament plasma would increase versus the gas pressure, leading to the air laser spatial distribution finally changing from a ring pattern to a donut pattern.

**Figure 6:**
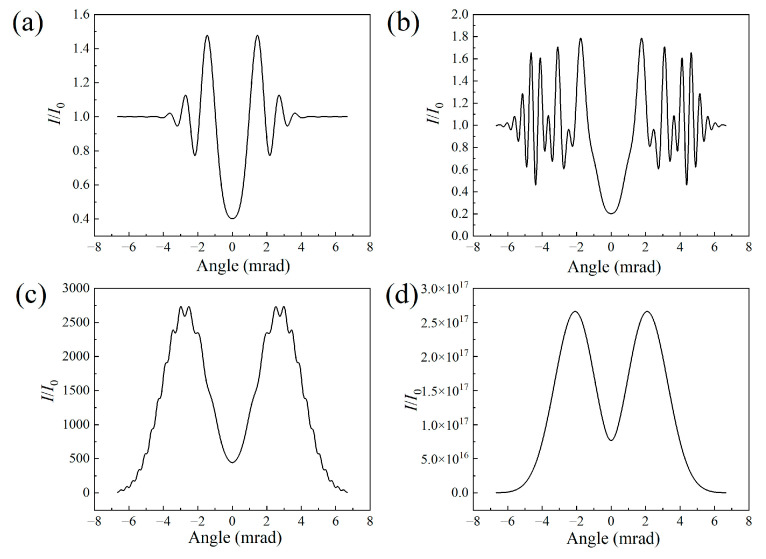
Angular distributions of air laser intensity for the nitrogen ion density of (**a**) 10^16^ cm^−3^, (**b**) 5 × 10^16^ cm^−3^, (**c**) 10^17^ cm^−3^, and (**d**) 2 × 10^17^ cm^−3^, respectively.

Figure 6 displays the air laser’s intensity as a function of the divergent angle for different peak nitrogen ion densities. Since increasing the nitrogen ion density would enhance the defocusing effect of the filament plasma, the outer rings get denser, with a strong diffraction effect. A typical donut-shaped pattern occurs as the outer ring signal merges into a smoothed-out line, as indicated in Figure 6d.

**Figure 7:**
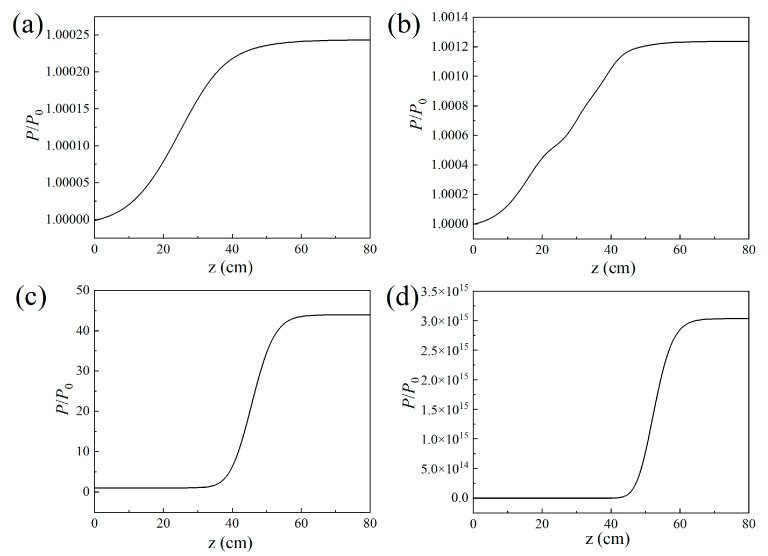
Air laser beam power along the propagation direction for peak nitrogen ion density of (**a**) 10^16^ cm^−3^, (**b**) 5 × 10^16^ cm^−3^, (**c**) 10^17^ cm^−3^, and (**d**) 2 × 10^17^ cm^−3^, respectively.

Further, the air laser’s beam power as a function of the propagation distance was recorded for different peak nitrogen ion densities, as shown in Figure 7. The laser’s beam power increases more significantly inside the laser filament plasma for a larger peak nitrogen ion density and remains almost constant after that. The fluctuations of the curve in Figure 7b indicate an interesting phenomenon, which can be clarified as follows: For the peak nitrogen ion density of 10^16^ cm^−3^, the results in Figure 5a1,b1 show that only several outer rings occur. Since the distance between the adjacent outer rings is so large that different outer rings will not intersect with each other. Thus, the air laser’s total power increases, smoothly propagating in the filament plasma induced by the lasing effect. For the peak nitrogen ion density of 5 × 10^16^ cm^−3^, the results in Figure 5a2,b2 show that the number of outer rings increases with the nitrogen ion density and the outer rings get denser. Due to the fact the adjacent outer rings are so close that they interfere or even intersect with each other, as shown in Figure 5a2, optical turbulence occurs. Therefore, the curve of the air laser’s power versus the propagation distance in the filament plasma shows fluctuations as depicted in Figure 7b. For a relatively large peak nitrogen ion density, notably thicker outer rings occur, which could be considered a donut-shaped pattern, as shown in Figure 5c,d. Under this condition, the optical turbulence disappears and the air laser’s power increases smoothly versus the propagation distance, as shown in Figure 7c.

In addition, the relationship between the gain effect and the peak nitrogen ion density was investigated, as indicated in Figure 4b. When the peak nitrogen ion density is larger than 10^17^ cm^−3^, the relationship between $\ln\left(P/P_{0}\right)$ and the peak nitrogen ion density can be fit linearly. This confirms that the gain coefficient of the air laser inside the filament increases with the nitrogen ion density. Moreover, the air laser’s beam power increases exponentially versus the peak nitrogen ion density.

Therefore, the results in Figure 4 meet well with the following equation for the air laser’s beam power propagating through the filament:

(4) $$P\alpha P_{0}\int_{0}^{L}e^{N_{e}(l)}dl$$

where *L* denotes the total length of the laser filament. $N_{e}(l)$ refers to the nitrogen ion density as a function of the propagation distance *l* inside the filament.

**Figure 8:**
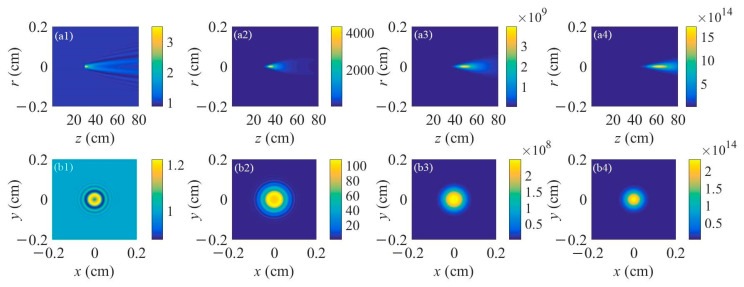
(**a1**–**a4**) Spatial distributions of the filament-induced nitrogen ion density versus propagation distance. (**a**) Spatial distributions of the air laser’s intensity versus propagation distance. (**b1**–**b4**) Transverse patterns of air laser intensity distribution on the cross-section at a propagation distance of 30 cm after the right end of the filament. The filament lengths are (**a1**,**b1**) 1 cm, (**a2**,**b2**) 10 cm, (**a3**,**b3**) 30 cm, and (**a4**,**b4**) 50 cm, respectively. The nonlinear refractive index term *n*_nl_ induced by the filament plasma defocusing effect was removed in the wave equation.

**Figure 9:**
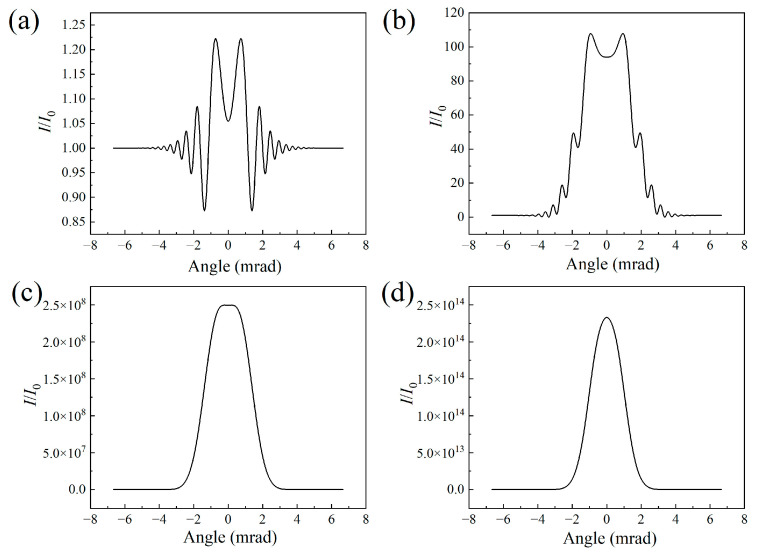
Angular distributions of the air laser’s intensity for filament lengths of (**a**) 1 cm, (**b**) 10 cm, (**c**) 30 cm, and (**d**) 50 cm. The nonlinear refractive index term *n*_nl_ induced by the filament plasma defocusing effect was removed in the wave equation.

**Figure 10:**
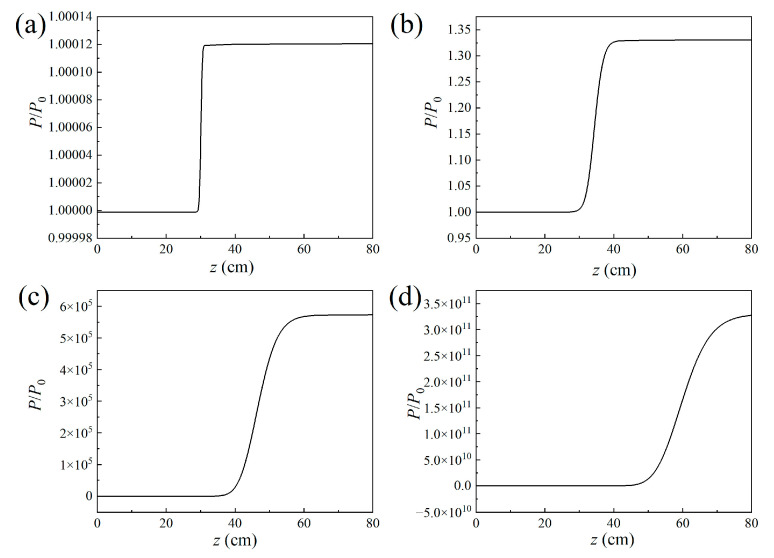
Air laser beam power along the propagation direction for the filament lengths of (**a**) 1 cm, (**b**) 10 cm, (**c**) 30 cm, and (**d**) 50 cm. The nonlinear refractive index term *n*_nl_ induced by the filament plasma defocusing effect was removed in the wave equation.

In order to investigate the influence of the filament plasma-induced defocusing effect on the spatial distribution of the air laser, the nonlinear refractive index term *n*_nl_ of the filament plasma was removed in Equation (1). The filament lengths are also chosen to be 1 cm, 10 cm, 30 cm, and 50 cm, while the peak nitrogen ion density is 10^17^ cm^−3^. The simulation results are shown in Figure 8, Figure 9 and Figure 10.

Figure 8a1–a4 shows the air laser’s intensity spatial distribution for different filament lengths. When compared with the results in Figure 1b1–b4, the peak intensity values get much larger without the *n*_nl_ term due to the continuous gain effect, without the divergence induced by the plasma inside the laser filament. Moreover, the air laser without the *n*_nl_ term diverges less significantly than that in Figure 1b1–b4 after the filament plasma. Figure 8b1–b4 depicts the intensity distribution on the cross-section of the air laser’s beam at the distance of 30 cm after the right end of the filament. Compared with Figure 1c1,c2 for the filament lengths of 1 cm and 10 cm, Figure 8b1,b2 indicates that the number of outer rings gets much smaller. Figure 8b3,b4 shows a Gaussian or super-Gaussian air laser beam intensity distribution without the outer ring or donut-shaped pattern induced by a relatively long filament. Therefore, the change of the air laser’s beam pattern from the outer rings pattern to a donut-shaped pattern with the increase of the filament lengths mainly results from the filament plasma defocusing effect.

Figure 9 shows the air laser intensity versus the divergent angle for different filament lengths. Figure 9a,b indicates that the air laser’s intensity on the central axis without the plasma defocusing item is much higher than that in Figure 2a,b, while the number of outer rings decreases a lot. Figure 9c depicts a super-Gaussian air laser beam profile for the filament length of 30 cm, while Figure 9d shows a Gaussian air laser beam profile for the filament length of 50 cm. With the increase of the filament length, the peak air laser intensity increases sharply without the plasma defocusing effect. Figure 10 depicts the air laser’s beam power versus the propagation distance. When compared with the results in Figure 3, it shows that the air laser’s total powers without the plasma defocusing item after the filament in Figure 10 are several orders higher than that with the plasma defocusing item for the filament lengths of 10 cm, 30 cm, and 50 cm.

## 4. Conclusions

In summary, the spatial intensity distribution of the air laser passing through the femtosecond laser filament has been investigated based on numerical simulation, which is seeded by an external plane continuous wave with a wavelength of 391 nm. The air laser beam pattern after the filament would vary from a ring structure to a donut shape by increasing the filament length or nitrogen ion density, mainly resulting from the plasma defocusing effect and the lasing effect inside the filament region. In addition, it has been demonstrated that the air laser’s beam power would increase exponentially versus the filament length and the nitrogen ion density. The knowledge about the angular distribution of air lasing could be important for optimizing the detection geometry of the LIDAR setup, including the view angle and the size of the collecting optical component.

## Data Availability

The data presented in this study are available on request.
